# Milk: Its Production and Uses

**Published:** 1904-06

**Authors:** 


**Milk: Its Production and Uses.** By EDWARD F. WILLOUGHBY, M.D., D.P.H. Pp. xii., 259. London: Charles Griffin & Co. Ltd. 1903.

Many of those who are engaged in the production, distribution, or control of milk must feel sufficient interest in their occupation to desire reliable information upon points not directly involved in their own particular duties, and the author of this small work has filled a distinct want in collecting within very reasonable limits practically all the available information likely to be of use to such a circle of readers. Without entering into such minute detail as would be serviceable only to the expert, Dr. Willoughby has succeeded in compiling a most readable account of the sources and uses of milk in which the ordinary inquirer will find a sufficient reply to most of the queries which suggest themselves to those who handle this important food in one capacity or another. The various breeds of cows and their relative merits, their housing, feeding, and the diseases to which they are subject, the composition and manipulation of milk and milk products, including fermented preparations, and, finally, the law relating to adulteration and the means of detecting sophistication are treated in a lucid, but on the whole technically correct manner.

In view of the probability of a call for further editions, it may be permitted to pay the greatest compliment open to the reviewer, and to point out one or two slips which detract from the merits of so excellent a work. On page 113 the milk of all mammalia is incorrectly stated to contain "ten to fourteen parts per thousand," instead of per cent., of solids. A somewhat awkwardly-expressed paragraph on p. 118 would lead many to infer that on drawing successive portions of milk from the bottom of a churn at rest, the first portions would contain more fat than the last, whereas the reverse is actually the fact, as is probably intended to be conveyed. It is hardly in accordance with established history to state that pathogenic germs are "mostly sporiferous," or that Pasteur was led to introduce the method of sterilising which bears his name by this consideration, or indeed that "Pasteurising" was invented or is now chiefly employed to eliminate pathogenic germs at all.

Among the tests for salicylic acid (p. 233), one which consists in the addition of bromine water with the production of a yellow, curdy precipitate and the perception of the "odour of a halogen" appears to be scarcely conclusive of the presence of this antiseptic, or indeed of much else than bromine.

Dr. Willoughby is to be commended for emphasising the view that the limits of composition adopted by the Board of Agriculture are not standards, but "the minimum permissible." When the magistrates who are called upon to decide cases of adulteration clearly understand and act upon this distinction less will be heard of the paltry fines commonly inflicted, and a further important step will have been taken towards the protection of the consumer.

